# Ghrelin Plasma Levels After 1 Year of Ketogenic Diet in Children With Refractory Epilepsy

**DOI:** 10.3389/fnut.2019.00112

**Published:** 2019-07-24

**Authors:** Maddalena Marchiò, Laura Roli, Chiara Lucchi, Anna Maria Costa, Matteo Borghi, Lorenzo Iughetti, Tommaso Trenti, Azzurra Guerra, Giuseppe Biagini

**Affiliations:** ^1^Laboratory of Experimental Epileptology, Department of Biomedical, Metabolic and Neural Sciences, University of Modena and Reggio Emilia, Modena, Italy; ^2^Pediatric Unit, Department of Medical and Surgical Sciences for Mothers, Children and Adults, University of Modena and Reggio Emilia, Modena, Italy; ^3^Department of Laboratory Medicine and Pathology, University Hospital and Local Healthcare Unit of Modena, Modena, Italy; ^4^Centro di Riferimento Oncologico, IRCCS, Aviano, Italy; ^5^Center for Neuroscience and Neurotechnology, University of Modena and Reggio Emilia, Modena, Italy

**Keywords:** children, epilepsy, ghrelin, growth, ketogenic diet

## Abstract

The ketogenic diet (KD) is a high-fat, low carbohydrate nutritional treatment adopted in several countries for refractory epilepsy. However, the use of KD is limited by adverse events including growth retardation. In a previous investigation, we demonstrated that ghrelin is reduced in children maintained on KD for 3 months. As ghrelin regulates growth hormone (GH) secretion, it can be hypothesized that growth retardation depends on the reduced ghrelin availability. To assess this hypothesis, in this study we evaluate ghrelin and growth during 1 year of KD. We examined a small cohort of 6 children (2 males and 4 females, age range 3–10.4 years) affected by refractory epilepsy, who received the KD as add-on treatment. All patients were on drug polytherapy. Endpoints of the study were: (i) ghrelin plasma levels at 0, 15, 30, 90, and 365 days from KD onset, (ii) growth, and (iii) seizure control by ketogenesis. Ghrelin levels were −53 and −47% of basal levels, respectively, at 90 and 365 days (*P* < 0.05 for both). Mean height index *z* scores were reduced, but not significantly, by comparing basal values with those at the end of observation. Instead, body mass index *z* scores slightly increased. Ketosis induced by the KD was within 2–5 mmol/L and satisfactorily reduced the seizure frequency (>50%) in all patients. We show that ghrelin plasma levels are consistently reduced in children with refractory epilepsy and maintained on the KD. This change was associated with low growth indexes in the majority of patients.

## Introduction

A high-fat, low-carbohydrate and normal protein diet has been developed, and named “ketogenic diet (KD),” to control antiseizure drug (ASD)-refractory epilepsy. This diet was designed to induce ketone body production, so to reproduce the physiological changes associated with prolonged fasting (1). In children with refractory seizures, the KD may be effective in up to 87% of cases (2). In spite of its demonstrated usefulness (3), various adverse events among which the most consistent is growth retardation (4–9) may limit the diffusion of KD as a medical nutritional treatment. Growth impairment in patients on the KD has been related to dysfunctional production of insulin-like growth factor-1 (IGF-1) (8), probably resulting from the alteration of mechanisms regulating growth hormone (GH) production. To this regard, the growth hormone secretagogue (GHS) ghrelin, which is a major regulator of food intake, metabolism, and GH secretion (10), could be responsible for the growth impairment and maybe for the reduction of IGF-1 observed in children on the KD.

Ghrelin is a peptide hormone prevalently released by gastric P/D1 cells and has also been involved in memory (11), anxiety (12), epilepsy (13), and neuroprotection (14). Interestingly, ghrelin is markedly dysregulated in pediatric patients with epilepsy and treated with ASDs (15). In addition, ghrelin has been associated with body weight gain in children with epilepsy and receiving valproate, thus suggesting an association between ghrelin and dysregulation of metabolism in epilepsy (16). In view of this evidence, we recently observed a time-dependent reduction in ghrelin plasma levels in children with refractory epilepsy and maintained on the KD (2). However, in these children we noticed a stable growth during the short 90 days' period of observation. For this reason, we decided to extend our previous study to establish if ghrelin levels could be stably reduced after 1 year of KD in children with refractory epilepsy, and the possible impact of the hypothesized ghrelin reduction on growth in the same patients.

## Materials and Methods

### Experimental Design

#### Patients

We considered a small cohort of 6 subjects composed by 2 males and 4 female children with diagnosis of refractory epilepsy, admitted to the pediatric neurology hospitalist service between October 2013 and June 2014. The inclusion criteria were: confirmed diagnosis of epilepsy, refractoriness to ASDs, age ranging from 0 to 14 years, and informed consent signed by parents. Whereas, exclusion criteria were acute or chronic metabolic diseases unrelated to epilepsy and the lack of adherence to the nutritional protocol. The KD was administered by starting with a 2:1 ratio of lipids and proteins + carbohydrates and, after few days, shifting to a 3.5/4:1 ratio as detailed previously (2, 17). KD was monitored and supplemented as previously described (2, 17). Height, weight and body mass index (BMI) were measured before the KD onset and until the end of observation period. Data about demographic features, clinical characteristics, diagnostic findings, therapeutic interventions, and clinical outcomes are reported in Table 1. The Ethics Committee of Modena (4206/C.E.) approved the research protocol according to local regulations and informed written consent was obtained from relatives of participants.

**Table 1:** Demographic and clinical features of patients treated with the ketogenic diet.

**Sex**	**Age**	**Age at epilepsy onset**	**Seizure type**	**Etiology**	**Concomitant ASDs**	**Type of KD**	**Reduction in seizure frequency (%)**	**β-hydroxybutyric acid (mmol/L, mean ± SEM)**	**Side effects**
F (1)	7.2	2.6	Spasm	Metabolic	CBZ, LEV, NZP	Classic 2:1	>75	3.9 ± 0.2	Hypercholesterolemia; Hyperoxaluria
F (2)	10.4	10.2	Spasm, Drop Attack, TC	Unknown	CBZ, LEV	Classic 4:1	>75	3.7 ± 0.1	Constipation; Vomiting
F (3)	4.2	4.2	Drop Attack, Myocl, Ab	Structural	LEV, TPM	Classic 2:1	>90	4.1 ± 0.1	Hypercholesterolemia
M (1)	5.2	2.3	TC, Drop Attack	Unknown	LEV, TPM	Classic 3:1	>90	3.3 ± 0.2	–
F (4)	5.2	5.2	TC, Drop Attack	Genetic	NZP, RFN, VPA	MCT 3.5:1	>90	3.6 ± 0.1	Nausea; Vomiting
M (2)	3	2.3	TC, Drop Attack	Unknown	TPX, VGB, VPA	Classic 4:1	>90	4.5 ± 0.2	–

*Ketosis levels were calculated by averaging all values obtained during each week. Ab, absence; ASDs, antiseizure drugs; CBZ, carbamazepine; KD, ketogenic diet; LEV, levetiracetam; Myocl, myoclonus; NZP, nitrazepam; RFN, rufinamide; SEM, standard error of the mean; TC, tonic-clonic; TPM, topiramate; VPA, valproate; VGB, vigabatrin*.

### Quantitative Analysis of Ghrelin

#### Reagents and Materials

To block conversion of ghrelin to des-acyl ghrelin (18), we used the protease inhibitor cocktail P2714 from Sigma Aldrich (Milan, Italy). The enzyme-linked immunosorbent assay (ELISA) kit for human plasma acyl ghrelin (EZGRA-88K) was obtained from Merck Millipore (Milan, Italy).

#### Sample Processing

Fasting blood samples (8:00–9:00 a.m.) were obtained (3 mL) and coded to assure a blind processing for immunoassays. Blood collected in tubes with dipotassium ethylenediaminetetraacetate dihydrate and 10% (v/v) P2714 was gently shaken and quickly placed on ice, then 15 min centrifuged (1,800 g at 4°C) to store plasma into sterile microtubes (200 μL) at −80°C. 4-(2-aminoethyl) benzenesulphonyl fluoride hydrochloride, contained in P2714, was previously shown to block ghrelin des-acylation effectively even when samples were kept frozen for months (18).

#### Immunoassays

Immunoassays were performed according to instructions. Samples were processed all together. Standards, controls, and coded samples were added to plates coated with the primary antibody and incubated at room temperature (RT) for 2 h. Then, plates were washed (300 μL to each well) five times and the enzyme added (100 μL) and incubated at RT for 2 h. After rewashing, substrate solution (200 μL) was dispensed to wells. Then, plates were gently shaken in the dark at RT for at least 30 min. Each well was mixed, and absorbance measured at 414 nm using a microplate reader (DTX 880 multimode detector, Beckman Coulter, USA). A cubic polynomial fitting was used to determine concentrations from the calibration curves. The intra-assay coefficient of variation (%) for ghrelin was 3.8, instead the inter-assay coefficient of variation was 7.5.

### Statistics

Data from immunoassays, height and body mass index values were all analyzed using one-way analysis of variance with repeated measures followed by Duncan's test for comparisons. All statistical analyses were performed using Sigmaplot 13 (Systat Software, San Jose, CA). Data are presented as mean ± standard error of the mean and regarded significantly different at *P* < 0.05.

## Results

We examined a small cohort of patients affected by refractory epilepsy, composed by 2 males and 4 female children. The mean age was 5.9 ± 1.1 year, ranging from 3.0 to 10.4 years. Demographic and clinical features of these children are illustrated in Table 1. In order to monitor the response to KD, patients' ketosis was daily assessed and ranged from 2 to 5 mmol/L. The reduction in seizure frequency obtained by administering the KD was satisfactory in all patients (>50%): 2 patients displayed more than 75% reduction, while the other 4 patients rarely presented seizures as recurrence was −90% of previous frequency. However, KD adjustments were required to obtain an effective control of seizures in the majority of children (Table 2). Nobody stopped to take ASDs and all were on polytherapy.

**Table 2:** Ketogenic diet (KD) was adapted to satisfy every specific requirement, as well as to obtain an adequate seizure control.

**Sex**	**Energy intake (kcal/day)**	**Lipids (%)**	**Proteins (%)**	**Carbohydrates (%)**	**Number of meals/day**	**Required adjustments**
F (1)	800	82	11	7	4	3
F (2)	1,600	89	8	3	4	2
F (3)	1,300	82	8	10	3	2
M (1)	1,250	87	5	8	4	4
F (4)	1,400	89	6	5	3	0
M (2)	1,350	90	8	2	3	3

*When seizure control was not satisfactory, the KD was adjusted. Only final KDs are reported in table*.

**Figure 1:**
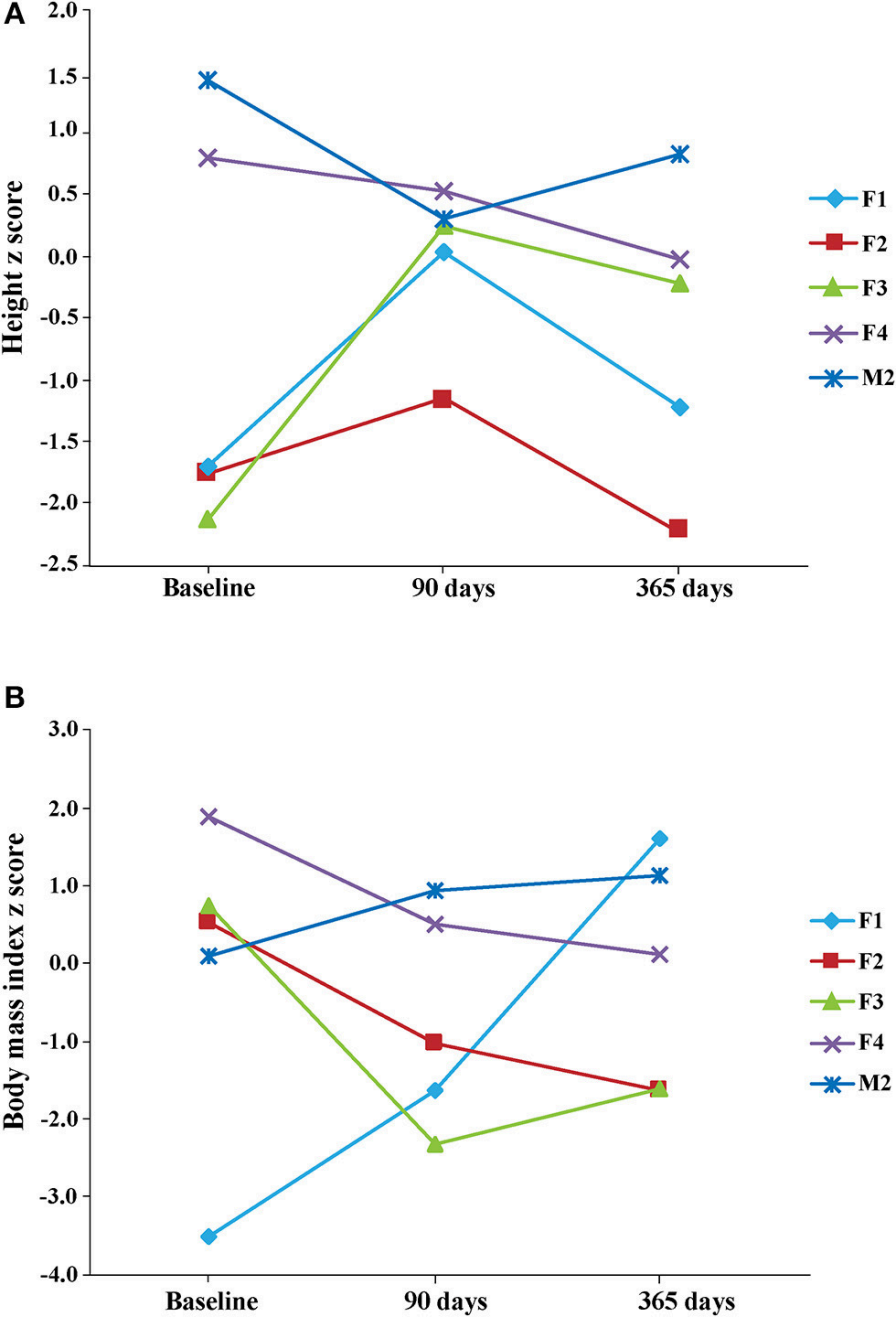
Height **(A)** and body mass index (BMI) **(B)** *z* scores were measured at the beginning of KD (baseline), after 3 months (90 days) and at the end of the observation period (365 days). Note that *z* scores were decreased in 3 patients at the end of the study, with respect to baseline scores. Height mean values and the respective standard deviation were: −0.670 ± 0.739 (*n* = 5) at baseline, 0.002 ± 0.295 (*n* = 5) 90 days later, and −1.198 ± 0.763 (*n* = 6) at the end of study. BMI mean values and the respective standard deviation were: 0.0560 ± 0.913 (*n* = 5) at baseline, −0.712 ± 0.624 (*n* = 5) 90 days later, and 0.155 ± 0.597 (*n* = 6) at the end of study. Note that one patient's data are not illustrated in the figure because height and BMI values were available only for the last time interval considered in our study.

Concerning growth, we calculated *z* scores immediately before the KD onset (−0.670 ± 0.739, *n* = 5), 90 days later (0.002 ± 0.295, *n* = 5), and at the end of our study (365 days; −1.198 ± 0.763, *n* = 6). Although we did not find significant differences, changes in the *z* score values were differently influenced by the KD timing (Figure 1). Specifically, height *z* scores were reduced in 3 out of 5 patients at the end of the study, and in 2 of them were even lower than those measured at beginning. Surprisingly, this change occurred after an initial increase of *z* scores observed at 90 days in 3 out of 5 children (Figure 1A). From 90 to 365 days, height *z* scores declined in 4 out of 5 children and only in one child this trend was the opposite. Concerning BMI *z* scores, a reduction was observed in 3 patients at the end of our study, whereas the other 2 presented a steady increase, at all considered time intervals (Figure 1B). Average values were: 0.0560 ± 0.913 prior to KD; −0.712 ± 0.624 at 90 days; 0.155 ± 0.597 at 365 days. All the observed changes did not seem to be related to differences in KDs, as illustrated in Table 2.

**Figure 2:**
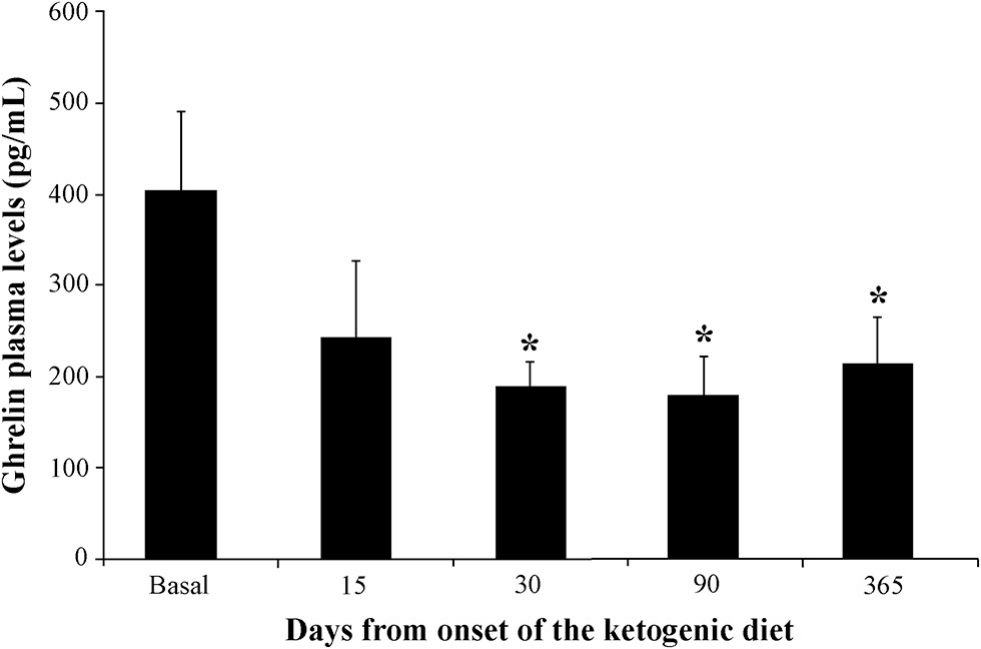
Changes in ghrelin plasma levels in children (*n* = 6) affected by refractory epilepsy and maintained on the ketogenic diet up to 1 year. *P < 0.05, one-way analysis of variance with repeated measures followed by Duncan's test.

Figure 2 illustrates ghrelin levels measured before and after the KD onset. Consistently, we observed stably reduced ghrelin plasma levels at 30 (−53%), 90 (−56%), and 365 (−47%) days after the KD onset. All reductions were statistically significant when compared with basal levels (*P* < 0.05 for all the above-mentioned time intervals, Duncan's test). It is worth mentioning that the reduction in ghrelin plasma levels was already detectable after 15 days (−40%), but it did not reach statistical significance because two samples were missing for technical reasons.

## Discussion

Growth impairment is an adverse event in children with refractory epilepsy receiving the KD. Although a study initially reported that growth was normal even after 6 months of KD (4), all subsequent studies demonstrated that weight and height were both compromised in the long-term (5–9). Interestingly, lack of changes at early time intervals was also confirmed by others who observed that growth impairment occurs only after 6 months of KD (7). In line with this report, we observed a non-significant change in growth after 3 months of KD (2), which is further confirmed by the present results. However, at the end of the study height *z* scores were more in line with the consistent findings reported by others (5–9).

The impact of KD on growth was initially related to the altered composition of the meal, especially for the reduction in micronutrients such as oligominerals and vitamins. However, authors excluded such an interpretation by comparing the effects on growth of the different KDs currently available to induce the ketosis (7). Also, the imbalance in protein-to-energy ratio has been suggested as responsible for the growth impairment (19). Therefore, we made any effort to carefully maintain a more than adequate caloric and protein intake, as well as in giving supplement vitamins and oligoelements to children, as previously described (2). For this reason, we are prone to believe that the malfunctioning of GH axis in children receiving the KD could explain the growth impairment. In line with this interpretation, we found that ghrelin is rapidly reduced and kept to approximately half basal values by the KD. After the KD onset, IGF-I is also immediately reduced and stabilized on low levels up to 1 year (8). As ghrelin is able to stimulate GH release, we suggest that the reduction in ghrelin levels may explain the reported reduction in IGF-I levels. To this regard, caution is needed because lack of any consequence for growth has been paradoxically found in animals in which ghrelin or its receptor was deleted (20). However, a missense mutation of GHS receptor segregating with short stature was reported for two unrelated families and, at least in one family, IGF-1 levels were markedly reduced (21). Consistently, growth velocity was increased in children of these families who received administration of GH.

The reason for this phenomenon could be the reduced gastric ghrelin expression and secretion observed in high-fat fed mice (22), suggesting that the same phenomenon may occur in patients receiving the KD. However, it has to be mentioned that also low carbohydrate high fat diets used for treatment of obesity have been found to modify ghrelin levels (23, 24). In these diets, the lipids and proteins + carbohydrates ratio is much lower than that recommended for refractory seizures. Moreover, restriction in energy intake is always present and adults are the main population involved in the studies, whereas KDs for epilepsy are usually proposed to children for whom no energy restriction is provided. So, the reported changes in ghrelin levels in response to reduction in carbohydrate intake may depend on factors different from those affecting ghrelin in children with epilepsy and on the KD. Additionally, results on changes in ghrelin level associated with reduction in carbohydrates intake were also controversial, since in one study ghrelin was reduced by 18% (23), whereas in another one an increase of 7% was observed (24). The magnitude of these changes was anyway not comparable to that found in our children, suggesting that the reduction in carbohydrates in KD does not completely explain our findings.

The reduction in ghrelin availability could result in other, less evident consequences apart from the growth impairment. Ghrelin has been identified as an anticonvulsant peptide (13). We recently found that ghrelin plasma levels are higher in children responding to ASDs, but not in those with refractory epilepsy (15). For this reason, the observed reduction in ghrelin availability due to KD may be unfavorable for the optimal control of seizures. Contrary to this hypothesis, in our children we observed a more than satisfactory reduction in seizure frequency. However, we suspect that the reduction in ghrelin levels may be involved in other phenomena. For instance, we described a paradoxical response in patients who developed increased seizure frequency and severity when starting the KD (17). To tentatively investigate this paradoxical response, in a seizure model we observed a prolongation of electrographic discharge induced by 6-Hz corneal stimulation in mice receiving the KD for a week. However, it remains to be established if ghrelin can play a role in these phenomena.

Ghrelin has also been demonstrated to possess protective properties in models of neuronal and vascular lesion [reviewed in Lucchi et al. (14)]. Indeed, ghrelin was found to attenuate kainic acid-induced neuronal cell loss in the mouse hippocampus (25). The ghrelin analog JMV-1843 rescued neurons and astrocytes in the pilocarpine model of *status epilepticus* (26). Beneficial effects were also reported for ghrelin in models of cerebral ischemia, although they have still to be clearly defined (27, 28). Interestingly, low ghrelin levels have been related to enhanced risk of liver damage in children undergoing surgery (29). This suggests that the reduction in ghrelin levels we observed in children with refractory epilepsy and receiving the KD may potentially expose to various consequences and should be addressed in order to prevent any of the possibly related adverse effects.

In conclusion, our study identified a long-lasting reduction in ghrelin levels in children with refractory epilepsy addressed by KD and receiving an adequate energy intake. The reduction in ghrelin availability could be related to the slower growth observed in children on KDs. However, a study based on a larger cohort is required to definitely demonstrate the association between the changes in ghrelin production and growth impairment due to the KD.

## Data Availability

All datasets generated for this study are included in the manuscript and/or the supplementary files.

## Ethics Statement

The Ethics Committee of Modena (4206/C.E.) approved the research protocol according to local regulations and informed written consent was obtained from relatives of participants.

## Author Contributions

GB: concept and design of the study. AG, CL, LR, MB, MM, and TT: data acquisition and analysis. AC, CL, GB, and LI: drafting the manuscript and figures. All authors read and approved the final version of the manuscript.

### Conflict of Interest Statement

The authors declare that the research was conducted in the absence of any commercial or financial relationships that could be construed as a potential conflict of interest.
